# Communication interventriculaire post infarctus du myocarde circonférentiel: à propos d'un cas et revue de la literature

**DOI:** 10.11604/pamj.2015.20.310.6271

**Published:** 2015-03-31

**Authors:** Ilham M'hamdi, Halima Benjelloune

**Affiliations:** 1 Service de Cardiologie A, CHU Avicenne, Rabat, Maroc

**Keywords:** Infarctus du myocarde, communication interventriculaire, facteurs prédictifs, myocardial infarction, ventricular septal defect, predictive factors

## Abstract

Malgré la réduction importante de la mortalité des infarctus aigus durant ces dernières décennies grâce a une prise en charge médicale adéquate; monitoring cardiaque, une reperfusion précoce; le taux de mortalité suite à une rupture du septum interventriculaire (communication interventriculaire CIV) reste considérable. Les facteurs de risques de cette complications a fait l'objet de plusieurs études: l'HTA, l’âge avancé, le sexe féminin, l'absence d'angine de poitrine et la localisation antérieure de l'ischémie. Les techniques de réparation chirurgicales ont évolué au fil du temps, mais le pronostic demeure très sombre avec un taux de mortalité inchangé depuis 1990. C'est pourquoi, il est très important d'en connaître les manifestations cliniques de façon à préciser le diagnostic par échocardiographie et permettre une prise en charge médico-chirurgicale urgente. Nous allons illustrer cette complication mortelle de l'infarctus du myocarde et mettre le point sur les différents facteurs prédictifs de son développement à travers un cas clinique et une revue de la littérature.

## Introduction

La communication interventriculaire (CIV) survenant après un infarctus du myocarde (IDM), est une complication rare mais redoutable, s'accompagnant d'un taux de mortalité très élevé (97% à 30 jours de l'IDM) [1]. Le pronostic s'améliore largement par la prise en charge chirurgicale. Nous allons mettre le point sur les différents facteurs prédictifs de son développement à travers un cas clinique et une revue de la littérature.

## Patient et observation

**Figure 1:**
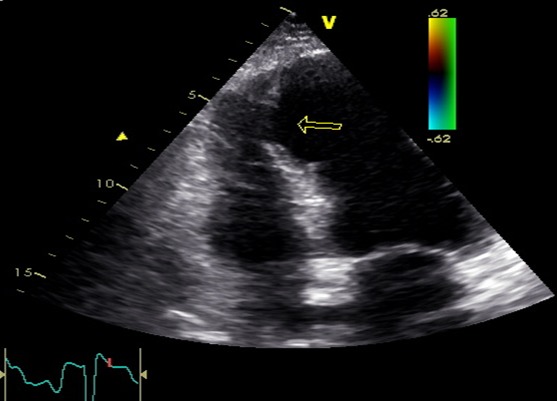
Aspect echocardiographique de la CIV

**Figure 2:**
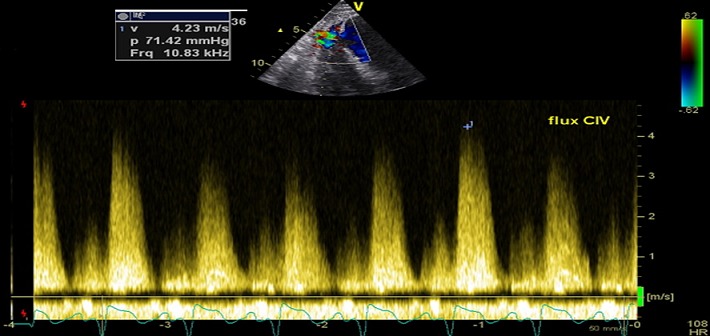
Aspect du flux de la CIV au Doppler continu

**Figure 3:**
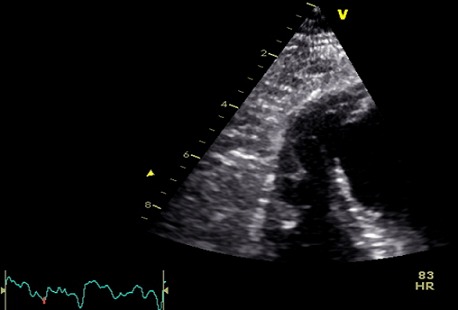
Aspect des trabéculations du VD colmatant la CIV

Nous rapportons le cas d'un patient de 64 ans, tabagique, diabétique, admis dans le service 15 jours après une scène infarctoide inaugurale, en rapport avec un IDM circonférentiel non thrombolysé, à l'admission le patient avait un pouls régulier à 85 bpm, une TA à 120/90 mmgh, une polypnée à 26c/ min, il n'avait pas de signes d'insuffisance cardiaque droite. L'auscultation cardio-pulmonaire a révélé la présence d'un souffle systolique irradiant en rayon de roue et des râles crépitant arrivant à mi champs. L’échocardiographie a objectivé une CIV musculaire de 15 mm inféro-septo-apicale (Figure 1), restrictive (Vmax à 4,10 m/s) (Figure 2), partiellement colmatée par les trabéculations du ventricule droit (Figure 3), un ventricule gauche (VG) non dilaté, siège d'une akinésie apicale et inféroseptale, en dysfonction systolique (FE= 47%). La coronarographie a montré des lésions bi-tronculaires (sténoses serrées sur l'interventriculaire antérieur (IVA) moyenne et distale, deux sténoses étagées courtes de la coronaire droite (CDT)). L’évolution a été marquée par le développement d'un anévrysme du VG. Après stabilisation par traitement médical, le patient a bénéficié d'une fermeture chirurgicale de la CIV, d'une cure de l'anévrysme ventriculaire gauche, d'un monopontage aortocoronaire sur la CDT, l'IVA étant prise par l'anévrysme qui suit son trajet. Les résultats ont été satisfaisants, avec une bonne évolution clinique.

## Discussion

A l'air préthrombolytique, l'incidence rapportée de la rupture septale était de 11% des séries anatomopathologiques, et de 2% des patients hospitalisés pour infarctus du myocarde. Actuellement elle n'est plus que de 0,2% [1, 2]. Et ce grâce aux différentes techniques de reperméabilisation précoce. D'autres facteurs prédictifs de cette complication ont été rapportés dans la littérature (Tableau 1). Comme l’âge avancé; en rapport avec la sénescence cardiaque et la perte de la capacité d'autoprotection du myocarde [3]. Le sexe féminin, jusqu´à maintenant le mécanisme expliquant cette prédominance féminine demeure inconnu, une hypothèse a été proposée, basée sur le concept d'avoir un collagène de structure plus susceptible à la rupture [3]. L'absence d'antécédents d'angor ou d'infarctus du myocarde [2, 4], expliquant le non développement de circulation collatérale, favorisant ainsi; surtout avec occlusion complète de l'artère responsable [2, 3]; la création d'une nécrose myocardique transmurale extensive exposant à la rupture septale.

**Tableau 1:** Les facteurs prédictifs de rupture septale après IDM retrouvés dans la littérature

GUSTO-I	STROCK	Anna et all
Age avancé	Age avancé	Age avancé > 70 ans
Sexe féminin	Sexe féminin	Sexe féminin
Infarctus antérieur	Infarctus inaugural	Infarctus antérieur
Absence de tabagisme	Sténose serrée TIMI 0/1	Lésion monotroculaire serré
		Faible indice de masse corporel
		Hypertrophie ventriculaire gauche

Dans l'essai GUSTO-I [1], il y avait une relation non linéaire entre les pressions systolique et diastolique au moment du recrutement, et l'incidence de la rupture septale. La corrélation positive (augmentation de l´incidence de la rupture septale avec l’élévation de la pression artérielle au dessus de 130/75 mmhg) reflète l'association entre l'hypertension artérielle et la rupture septale. Il a été constaté également que le tabagisme est un facteur protecteur contre cette complication. Une fréquence plus élevée a été notée avec les IDM de localisation antérieur [1, 3] Notre cas, n'illustre pas un cas typique des facteurs prédictifs de la CIV, puisqu'il présente des éléments qui divergent et d'autres qui convergent avec ce qui a été rapporté dans la littérature. C'est un homme de 61 ans, non hypertendu, qui a comme facteur protecteur le tabagisme. Par ailleurs il a présenté de façon inaugurale un infarctus du myocarde circonférentiel, sans bénéficier d'une reperfusion précoce.

## Conclusion

Malgré la diversité des facteurs prédictifs de la rupture septale, le principal facteur demeure l'absence de reperfusion précoce. D'ou l'intérêt d'optimiser la prise en charge à la phase aigue de l'IDM. Des séquelles fonctionnelles graves peuvent être évitées grâce au sauvetage myocardique précoce

## References

[1] Crenshaw BS, Granger CB, Birnbaum Y, Pieper KS, Morris DC, Kleiman NS (2000). Risk factors, angiographic patterns, and outcomes in patients with ventricular septal defect complicating acute myocardial infarction; GUSTO-I (Global Utilization of Streptokinase and TPA for Occluded Coronary Arteries) Trial Investigators. Circulation..

[2] Menon V, Webb JG, Hillis LD, Sleeper LA, Abboud R, Dzavik V (2000). Outcome and profile of ventricular septal rupture with cardiogenic shock after myocardial infarction: a report from the SHOCK Trial Registry: Should we emergently revascularize Occluded Coronaries in cardiogenic shocK?. J Am Coll Cardiol..

[3] Ledakowicz-Polak A, Polak L, Zielinskra M (2011). Ventricular septal defect complicating acute myocardial infarction-still an unsolved problem in the invasive treatment era. Cardiovasc Pathol..

[4] Lazopoulos G, Manns-kantartzis M, Kantartzis (2009). Giant Left Ventricular Aneurysm and Intraventricular Septal Defect After Silent Myocardial Infarction. Hellenic J Cardiol..

